# Management of stage 1 TTR FAP: French experience

**DOI:** 10.1186/1750-1172-10-S1-P65

**Published:** 2015-11-02

**Authors:** David Adams, Marie Théaudin, Pierre Lozeron, Clovis Adam, Guillemette Beaudonnet, Céline Labeyrie, Catherine Lacroix, Cécile Cauquil

**Affiliations:** 1 CHU Bicêtre, APHP, French Reference Center for FAP (NNERF), Univ Paris Sud, INSERM U1191, Le Kremlin Bicêtre, Paris, France

## Introduction

Transthyretin Familial Amyloid Polyneuropathy (TTR-FAP) is a worldwide autosomal dominant disease due to point mutation of TTR gene. The main endemic area is Portugal, associated with V30M variant and an early onset (EO) (mean age 30 yo). Phenotype is a progressive length dependent small fiber polyneuropathy with autonomic dysfunction and cardiac conduction disorders; the median survival is 12 years. France is a prototypic non endemic country, characterized by sporadic cases in 50%, a late onset (LO>50 yo) in 75%, a genetic heterogeneity (41 variants ; V30M in 55%). Liver transplantation (LT) is the gold standard treatment allows to stop progression of the neuropathy in EO V30M patients and to prolonge significantly the survival. Tafamidis, a TTR kinetic stabilizer, received marketing authorization in Europe for stage 1 FAP (walking unaided) allowing to slow progression of the neuropathy. Aims of the study was to assess the place of tafamidis in the management of TTR-FAP.

## Methods

In a population of 131 patients diagnosed during period (2008-2012). Mean age : 59.3 yo SD 15.9. Stage 1 (60%) stage 2 (36%) stage 3 (4%). Mutation : V30M: 59%, non V30M variants n=18, Late onset (LO): 73.3%. mean NIS : 40.65 SD 28.6. In Stage 1 mean NIS=27.32 SD 20. Locomotion (PND score) : 71% with Walking difficulties. Patients were followed periodically (every 6 months) in consultation assessing by a questionnaire : new manifestations (sensory complaints, walking difficulties, autonomic dysfunction (digestive (gastroparesia, diarrhea), erectile dysfunction)). And on examination looking for orthostatic dysfunction.

## Results

Among the 78 patients of stage 1, 56 accessed to tafamidis (73% V30MTTR), 16 underwent LT, 6 were lost of follow-up. The follow-up ranged from 0.5-4.5 years. Fourteen patients (25%) remained stable for > 2 years under tafamidis (max 4.5 years) including 8/15 with NIS<10, 80% V30MTTR; 7/15 EO. Twenty three/56 pts (41%) worsened with increased or onset walking disability (n=14); daily diarrhea (5) with anal incontinence (3); extensive painful and sensory neuropathy (n=2), hand weakness (n=1), onset of erectile dysfunction (n=1). Worsening occurred during the first year in 65%, between 1 to 2.5 years in 35%. Most of them had a NIS ≥10 (87%), 69% V30MTTR; 48% LO. Among the 23 patients who worsened, 12 were enrolled in clinical trials with TTR gene silencing ; 11 underwent LT.

## Conclusions

Treatment of stage 1 TTR-FAP by tafamidis requires a close and long term monitoring of patients including a detailed questionnaire on walking disability, autonomic dysfunction, extension of pain and sensory loss. In case of significant progression in these items, a switch to clinical trials or LT should be done.

